# Supplementary material for: Complexome profiling of the Chlamydomonas psb28 mutant reveals TEF5 as an early PSII assembly factor
Source: Plant Cell. 2025 Mar 17;37(6):koaf055. doi: 10.1093/plcell/koaf055 (PMC12164590; doi:10.1093/plcell/koaf055)
Supplement: koaf055_Supplementary_Data [file koaf055_supplementary_data.zip › psb28_interactive.zip!/plots/intensity/qualityPlots/peptides/PCY1_Cre03.g182551.t1.2.html]

### Cre03.g182551.t1.2

Trivial name: PCY1  
SeqCoverage [median]: 9.00  
Mass: 14.88 kDa  
  
 

| Sequence | MissedCleavage | Mass [Da] | wtIntensity | muIntensity | Score |
| --- | --- | --- | --- | --- | --- |
| LGADSGALEFVPK | 0 | 1302.68 | 4013.09 | 0.00 | 177.44 |
|  |  |  | 4013.1 | 0.0 |  |
